# A chromosome-level genome for the flower thrips *Frankliniella intonsa*

**DOI:** 10.1038/s41597-024-03113-6

**Published:** 2024-03-08

**Authors:** Wei Song, Jia-Xu Wang, Li-Jun Cao, Jin-Cui Chen, Wen-Xue Bao, Min Chen, Shu-Jun Wei

**Affiliations:** 1 Institute of Plant Protection, Beijing Academy of Agriculture and Forestry Sciences, Beijing, 100097 China; 2 Beijing Key Laboratory for Forest Pests Control, Beijing Forestry University, Beijing, 100083 China; 3 College of Forestry, Inner Mongolia Agricultural University, Hohhot, 010019 China

**Keywords:** Entomology, Genomics

## Abstract

The flower thrips *Frankliniella intonsa* (Thysanoptera: Thripidae) is a common insect found in flowers of many plants. Sometimes, *F. intonsa* causes damage to crops through direct feeding and transmission of plant viruses. Here, we assembled a chromosomal level genome of *F. intonsa* using the Illumina, Oxford Nanopore (ONT), and Hi-C technologies. The assembled genome had a size of 209.09 Mb, with a contig N50 of 997 bp, scaffold N50 of 13.415 Mb, and BUSCO completeness of 92.5%. The assembled contigs were anchored on 15 chromosomes. A set of 14,109 protein-coding genes were annotated in the genome with a BUSCO completeness of 95.0%. The genome contained 491 non-coding RNA and 0.57% of interspersed repeats. This high-quality genome provides a valuable resource for understanding the ecology, genetics, and evolution of *F. intonsa*, as well as for controlling thrips pests.

## Background & Summary

Thrips are small insects from the order Thysanoptera. Among the currently described thrips, only about 150 species are recognized as pests^1^. The flower thrips *Frankliniella intonsa* is a common species found in flowers of many plants. It is native to Eurasia, but now introduced to Oceania and North America^2–6^. Despite their small body size allowing for easy dispersal, the distribution of *F. intonsa* remains limited compared to a cosmopolitan pest from the same genus, the western flower thrips, *Frankliniella occidentalis*^7–9^. In its native range, *F. intonsa* was reported as a pest at times^10^ but often found alongside other thrips in the field, leading to species competition and displacement^11–15^. However, in recent years, *F. intonsa* has been more frequently treated as a pest of crops^13,16^. In some regions, *F. intonsa* has developed resistance to insecticides used for its control^17,18^. In addition, *F. intonsa* has been found as a vector of plant virus from the genus *Tospovirus*^19–21^, although its transmission efficacy is lower than *F. occidentalis*^11^. Therefore, we need to understand its biology, ecology, and evolution, as well as its competition with other species, to reassess the pest status of *F. intonsa* and develop a proper control strategy^22,23^. Well-assembled genomes will provide genetic resources for the study of *F. intonsa*. Currently, genomes of thrips have been reported for the western flower thrips *Frankliniella occidentalis*^24^, tobacco thrips *Frankliniella fusca*^25^, melon thrips *Thrips palmi*^26^, bean flower thrips *Megalurothrips usitatus*^27,28^ and rice thrips *Stenchaetothrips biformis*^29^. Recently, a parallel study of ours published a genome for *F. intonsa* that represents the first chromosome-scale genome for the species of the genus *Frankliniella*^30^. The specimens used for *F. intonsa* genome sequencing were collected from Zhejiang Province of southern China^30^. Here, we assembled another chromosome-level genome for *F. intonsa*, which was sequenced from specimens collected from Inner Mongolia of northern China, to enrich the genetic resources of this species. We utilized Illumina short-read sequences to estimate the genome features of *F. intonsa*. We also employed Oxford Nanopore Technologies (ONT) long-read sequences to assemble a contig-level genome. Furthermore, we utilized chromosome conformation capture (Hi-C) technology to assemble these contigs into a chromosome-level genome.

## Methods

### Sample collection and genomic DNA sequencing

A strain used for genome sequencing was reared for 10 generations in the laboratory at the College of Forestry, Inner Mongolia Agricultural University, Hohhot, China. About 100 unsexed adults collected from Huanghuagou Scenic Area in Chaha’er Right Wing Central Banner, Inner Mongolia, China (E 112°32′03″, N 41°08′17″) were used to establish the strain. *Frankliniella intonsa* was reared on the seedling of horsebean *Vicia faba* under the following laboratory conditions: 25 °C, 60% relative humidity and a 16 L:8D photoperiod. The specimens used for sequencing were morphologically identified to avoid the inclusion of other thrips species. About 1,000 adults with pooled male and female samples were utilized for the extraction of high-molecular-weight DNA (HMW DNA) and subsequent library construction. Genomic DNA was extracted from the entire body of pooled individuals using the Qiagen MagAttract HMW DNA Mini Kit, following the manufacturer’s protocol. A short-read DNA library with an insert size of 500 bp was constructed using the Illumina TruSeq DNA PCR-Free HT LPK and sequenced on the Illumina X Ten platforms (Illumina Inc., San Diego, CA, USA). A long-read DNA library with an insert size of 23 kb was prepared according to the manufacturer’s protocol and sequenced using the PromethION model of the ONT platform. The short reads were used for genome survey analysis, including estimating the genome size, and rates of heterozygosity and duplication, as well as for correcting the assembly from the long sequencing reads, while the long reads were used for the contig-level genome assembly. The sequencing process generated 15.55 Gb (73.88X coverage) of clean short-read data and 28.35 Gb (135.65X coverage) of long-read data, respectively (Table 1).

**Table 1:** Sequencing data generated in this study for genome assembly of *Frankliniella intonsa*.

Library	Insert size	Data (Gb)	Coverage (X)	Usage
Illumina	500 bp	15.44	73.88	Genome survey
ONT	23 kb	28.35	135.65	Scaffold assembly
Hi-C	100–500 bp	26.97	129.04	Chromosome assembly

### Hi-C library construction and sequencing

The chromosome conformation of the genome was captured to determine the order and orientation of the contigs. Approximately 1,000 adults of mixed sex were used for constructing the Hi-C library. The specimens were ground and then cross-linked in a fresh, ice-cold nuclear isolation buffer with a 2% formaldehyde solution for 10 minutes at room temperature. The fixed cells were digested using *DpnII* (NEB) enzymes and processed according to the standard operating procedure for Hi-C library construction, which included cell lysis, incubation, labelling the DNA ends with biotin-14-dCTP, and performing blunt-end ligation of crosslinked fragments. The Hi-C library was amplified by 12–14 PCR cycles and sequenced on the Illumina NovaSeq. 6000 platform. A total of 26.97 Gb of clean data were generated, representing 120.05X coverage of the genome (Table 1).

### Genome characteristics estimation

**Fig. 1:**
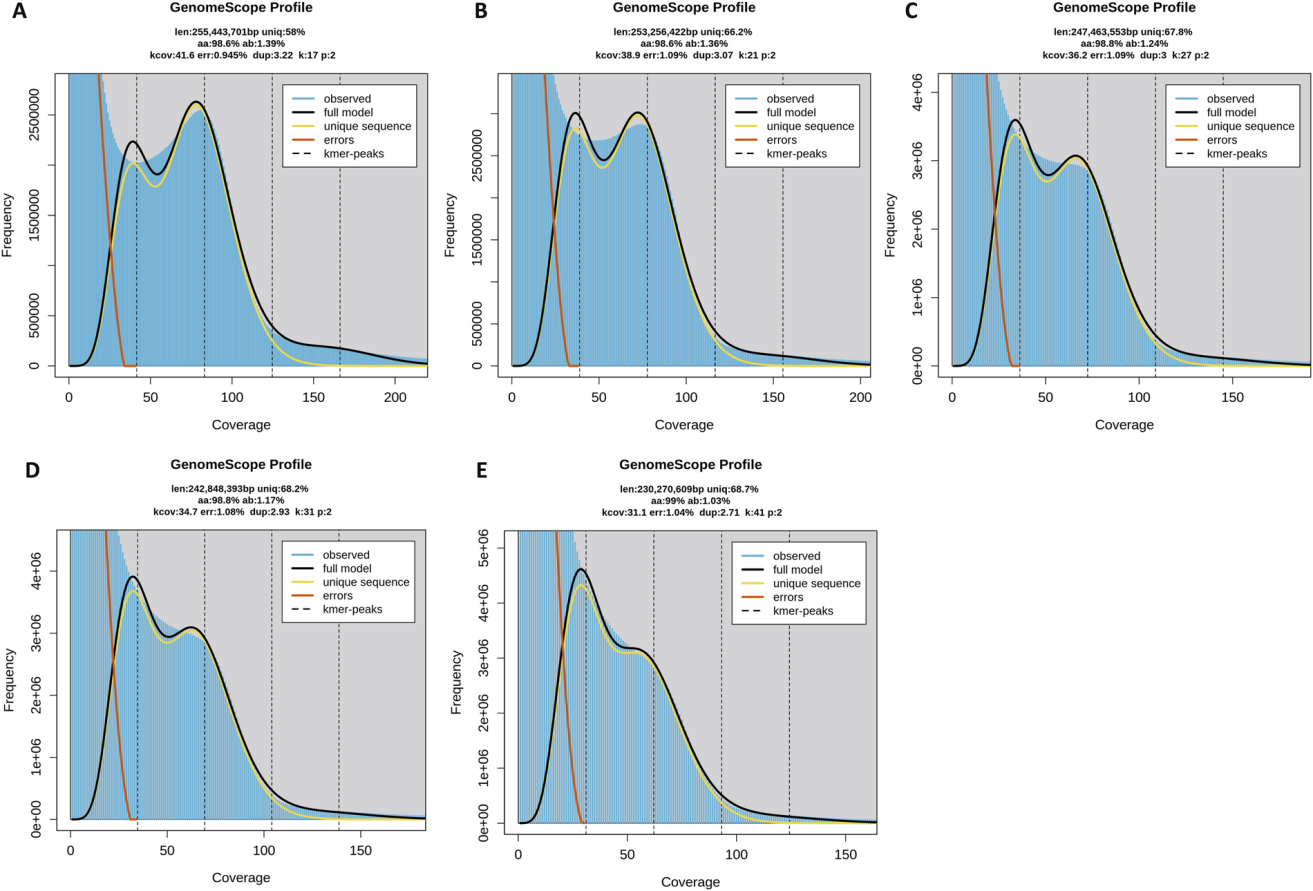
Estimated characteristics of *Frankliniella intonsa* genome based on Illumina short-read data. Results were obtained in GenomeScope version 2.0 with 17- (**A**), 21- (**B**), 27- (**C**), 31- (**D**) and 41- (**E**) mer. The *K*-mer distributions showed double peaks: the first peak indicates genome duplication and the highest peak represents a genome size peak. len, estimated genome size in bp; aa, homozygosity rate; ab, heterozygosity rate; dup, duplication rate.

Genome characteristics were estimated based on Illumina short-reads. The raw sequences were trimmed using the software fastp^31^ under the default parameters. KMC version 3.0^32^ was used to count the *K*-mer distribution histogram under 17, 21, 27, 31 and 41-mer with parameters ‘-m96 -ci1 -cs10000’ and ‘-cx10000’, based on the trimmed data. The genome size, heterozygosity rate, and duplication rate were estimated using GCE version 2.0 under the default parameters^33^. The estimated genome size decreased as the *K*-mer increased, ranging from 230 Mb to 255 Mb, similar to a previous study of this species^30^. The genome duplication decreased as the *K*-mer increased, with values ranging from 2.71% to 3.22%, higher than a previous study of this species (2.04%)^30^. Each *K*-mer distribution showed double-peaks, indicating a highly complex genome (Table 2, Fig. 1).

**Table 2:** Statistics for chromosomal-level assembly and annotation of *Frankliniella intonsa* genome.

Type	Item	Feature
Genome survey	Genome size (Mb)	230.27–255.44
	Error rate	0.945%–1.09%
	Duplicated sequence	2.71%–3.22%
Genome feature	Genome size (Mb)	209.09
	Chromosome number	15
	Contig number	422839
	Longest scaffold (Mb)	21.406
	Shortest scaffold (Mb)	10.106
	Rate of N	10.71%
	GC content	51.75%
	Scaffold N50 (Mb)	13.415
	Contig N50 (bp)	995
	BUSCO completeness	C:95.0% [S:94.4%, D:0.6%], F:0.4%, M:4.6%, n:1367
Protein-coding gene	Gene number	14109
	BUSCO completeness	C:95.2% [S:94.2%, D:1.0%], F:0.4%, M:4.4%, n:1367
	Functional annotation	9931

### Genome assembly and annotation

**Fig. 2:**
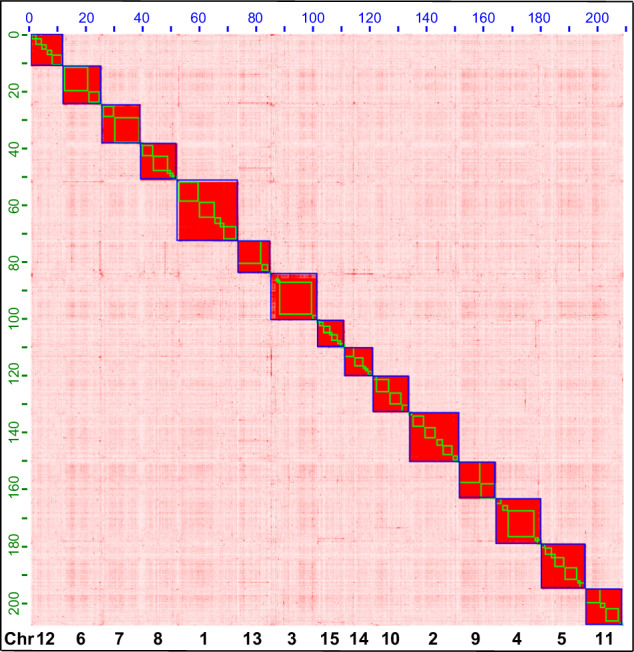
Genome-wide contact matrix of *Frankliniella intonsa* generated using Hi-C data. Each blue square represents a chromosome, each green square represents a contig. Fifteen chromosomes were anchored under the default parameters of Juicer and 3D-DNA software. Numbers on the top and left axes show the chromosome length in Mb, numbers on the bottom axes show the chromosome number. Chromosomes are numbered based on their size, from the largest to the smallest.

The long-reads from ONT were quality-controlled and assembled into contigs using a “correct-then-assemble” strategy in nextDenovo version 2.5.2^34^ with parameters ‘read_cutoff = 1k, genome_size = 400 m, pa_correction = 20, sort_options = -m 100 g -t 10, minimap2_options_raw = -t 10, correction_options = -p 15, minimap2_options_cns = -t 10, nextgraph_options = -a 1’. These contigs were then polished three times based on the Illumina short reads using pilon version 1.22^35^ under the default parameters. The polished contigs were further assembled into a chromosomal-level genome using Hi-C sequencing data. Low-quality reads and adapters from the Hi-C library were filtered using Trimmomatic version 0.39^36^ under the default parameters and then mapped to the assembled contigs using Juicer^37^ with default parameters. The reads were grouped into chromosomes using 3D de novo assembly (3D-DNA) version 180922 with parameters ‘–editor_repeat_coverage = 15, -r 2’^38^. Mistakes were manually adjusted in Juicebox version 2.16.00 (https://github.com/aidenlab/Juicebox), and the raw-chromosomes were updated using the script “run-asm-pipeline-post-review.sh” in 3D-DNA again. At last, the repeat-masked high-quality genome assembly was submitted to the online tool Helixer^39^ under the invertebrate mode for genome structure annotation. Functional annotation was performed by blasting the proteins against the Uniport/SwissProt database using blastp version 2.12.0+^40^ under the following parameters: ‘-evalue 0.000001 -outfmt 6 -num_threads 128 -num_alignments 1 -seg yes -soft_masking true -lcase_masking -max_hsps 1’. In total 422,839 contigs were assembled into 15 chromosomes (Fig. 2). The largest chromosome size was 21.406 Mb and the shortest was 10.106 Mb. We numbered the chromosomes in descending order of their size. The total length of the anchored genome was 209.09 Mb with an N50 of 13.415 Mb. About 57 Mb contigs were not anchored to any chromosome. The anchored genome size is shorter than the estimated genome size and a previously assembled genome for this species^30^. Both anchored and unanchored contigs were submitted to GenBank with accession numbers CM069028.1- CM069042.1. In total, 14,109 protein-coding genes (PCGs) were identified with 9,931 genes have functional annotation^41^. The G + C content of the final genome assembly was 51.75% (Table 2).

### Repeat elements and non-coding RNA predictions

The repetitive elements longer than 1000 bp were identified against the Insecta repeats within RepBase Update (20120418). The identification was performed using RepeatMasker version open-4.0.0^42^ (-no_is -norna -xsmall -q) with the search engine RM-BLAST (v2.2.23+). *De novo* identification of transposable elements (TEs) was performed using RepeatModeler^43^. Non-coding RNAs were identified using Rfam^44,45^, while ribosome RNAs (rRNAs) and transfer RNAs (tRNAs) were searched by tRNAscan-SE v2.0^46^ and RNAmmer v1.2^47^, both under default parameters. A total of 393,270 transposable elements (TEs) were identified, including 3,903 retroelements with a total length of 452,458 bp, 8,858 DNA transposons and 380,509 Tandem Repeats (TRs) (Table 3). We identified 48 miRNAs, 87 snRNAs, 30 snoRNAs, 143 rRNAs and 183 tRNAs in *F. intonsa* genome (Table 4).

**Table 3:** Repeated elements identified in the *Frankliniella intonsa* genome.

Element class	No. element	Length (bp)	Percentage (%)
Retroelements	3903	452458	0.22
SINEs	68	4413	0
Penelope	341	18665	0.01
LINEs	1582	162676	0.08
L2/CR1/Rex	309	44465	0.02
R1/LOA/Jockey	379	37449	0.02
R2/R4/NeSL	15	2711	0
RTE/Bov-B	25	1699	0
L1/CIN4	12	729	0
LTR elements	2253	285369	0.14
BEL/Pao	160	12254	0.01
Ty1/Copia	910	143086	0.07
Gypsy/DIRS1	1160	128160	0.06
DNA transposons	8858	622062	0.3
Unclassified	1470	127069	0.06
Total interspersed repeats		1201589	0.57
Small RNA	100	9929	0
Satellites	53	8240	0
Simple repeats	380456	18406334	8.8
Low complexity	46414	2608334	1.25

**Table 4:** Non-coding RNA identified in the *Frankliniella intonsa* genome.

Class		Number
miRNA Count		48
snRNA Count		87
snoRNA Count		30
rRNA count	5s_rRNA	58
	5_8s_rRNA	1
	8s_rRNA	84
tRNA count	tRNAs decoding Standard 20 AA	157
	Selenocysteine tRNAs (TCA)	1
	Possible suppressor tRNAs (CTA, TTA)	0
	tRNAs with undetermined/ unknown isotypes	1
	Predicted pseudogenes	24
	Total tRNAs	183
	tRNAs with intron	12

## Data Records

The genome project was deposited at NCBI under BioProject No. PRJNA1016113. Genomic Illumina sequencing data were deposited in the Sequence Read Archive at NCBI under accession SRR26105494^48^. Genomic ONT sequencing data were deposited in the Sequence Read Archive at NCBI under accession SRP461583^49^. The Hi-C sequencing data were deposited in the Sequence Read Archive at NCBI under accession SRR26122928^50^. The genome assembly, genome annotation, and protein coding genes files were deposited in Figshare under a DOI of 10.6084/m9.figshare.24174591.v5^41^. The final genome assembly was also deposited in GenBank at NCBI under the accession number GCA_035584235.1^51^.

## Technical Validation

The extracted high molecular weight (HMW) DNA had an average size of approximately 23 Kb, as determined by pulsed-field gel electrophoresis. To assess the integrity and quality of the genome assembly and the set of protein-coding genes, Benchmarking Universal Single-Copy Orthologs (BUSCO) version 5.4.5^52^ was used. For the chromosome-level genome assembly, the BUSCO completeness was 93.3%, 95.6%, 96.1% and 95.0%, based on the Eukaryota, Metazoa, Arthropoda and Insecta (odb_10, released on 2024-01-08) datasets, while the previously assembled genome has a completeness of 96.9%–98.8%^30^. For the protein-coding gene set, the BUSCO completeness was 93.0%, 94.6%, 96.3% and 95.2% based on the Eukaryota, Metazoa, Arthropoda and Insecta datasets, respectively, while the previously assembled genome has a completeness of 89.5%–94.4%^30^. We mapped our Illumina short-read to the assembled genomes using BWA version 0.7.17-r1198-dirty^53^ under the BWA-MEM algorithm. The mapping rate of short-reads data to our unmasked chromosomal-level genome and that of Zhang *et al*.^30^ is 81.92% and 87.30%, respectively. We also mapped the Illumina short-read of Zhang *et al*.^30^ and obtained a mapping rate of 84.04% for our genome assembly and 92.80% for the assembly of Zhang *et al*.^30^.

## Data Availability

No specific code or script were used in this study.

## References

[1] Mound LA, Wang Z, Lima EFB, Marullo R (2022). Problems with the concept of “Pest” among the diversity of pestiferous Thrips. Insects.

[2] Zur Strassen, R. *Die terebranten thysanopteren Europas und des mittelmeer-gebietes*. (Goecke & Evers, 2003).

[3] Nakahara, S. & Foottit, R. G. *Frankliniella intonsa* (Trybom) (Thysanoptera: Thripidae), an invasive insect in North America. *P. Entomol. Soc. Wash*. (2007).

[4] Teulon D, Nielsen M (2005). Distribution of western (glasshouse strain) and intonsa flower thrips in New Zealand. New Zealand Plant Protection.

[5] Wang Z, Mound L, Tong X (2019). Phylogenetic relationships within the *Frankliniella* genus-group based on morphology, with a revision of Iridothrips (Thysanoptera, Thripidae). Zootaxa.

[6] Wang CL, Lin FC, Chiu YC, Shih HT (2010). Species of *Frankliniella* Trybom (Thysanoptera: Thripidae) from the Asian-Pacific Area. Zool. Stud..

[7] Reitz SR (2009). Biology and ecology of the western flower thrips (Thysanoptera: Thripidae): the making of a pest. Fla. Entomol..

[8] Morse JG, Hoddle MS (2006). Invasion biology of thrips. Annu. Rev. Entomol..

[9] Reitz SR (2020). Invasion biology, ecology, and management of western flower thrips. Annu. Rev. Entomol..

[10] Kijima K, Ohno S, Ganaha-Kikumura T, Shimizu T (2013). Control of flower thrips, *Flankliniella intonsa* (Trybom) and sweetpotato whitefly, *Bemisia tabaci* (Gennadius) on sweet pepper in greenhouses in Okinawa, Southwestern Japan, by releasing polyphagous indigenous predator, *Campylomma chinensis* Schuh (Hemiptera: Miridae). Jpn J. Appl. Entomol. Z..

[11] Okuda S, Okuda M, Matsuura S, Okazaki S, Iwai H (2013). Competence of *Frankliniella occidentalis* and *Frankliniella intonsa* strains as vectors for *Chrysanthemum stem necrosis virus*. Eur. J. Plant Pathol..

[12] Bhuyain MMH, Lim UT (2020). Relative susceptibility to pesticides and environmental conditions of *Frankliniella intonsa* and *F. occidentalis* (Thysanoptera: Thripidae), an underlying reason for their asymmetrical occurrence. PloS ONE.

[13] Fu B (2022). Spinetoram resistance drives interspecific competition between *Megalurothrips usitatus* and *Frankliniella intonsa*. Pest Manag. Sci..

[14] Pobozniak M (2011). The occurrence of thrips (Thysanoptera) on food legumes (Fabaceae). J. Plant Dis. Protect..

[15] Alim MA, Song J, Seo HJ, Choi JJ (2018). Monitoring thrips species with yellow sticky traps in astringent persimmon orchards in Korea. Appl. Entomol. Zool..

[16] Tang LD, Guo LH, Shen Z, Chen YM, Zang LS (2023). Comparison of the biology of *Frankliniella intonsa* and *Megalurothrips usitatus* on cowpea pods under natural regimes through an age-stage, two-sex life table approach. Bull. Entomol. Res..

[17] Hiruta, E., Aizawa, M., Nakano, A. & Sonoda, S. Nicotinic acetylcholine receptor *α6* subunit mutation (G275V) found in a spinosad-resistant strain of the flower thrips, *Frankliniella intonsa* (Thysanoptera: Thripidae). *J. Pestic. Sci*. **43**, D18-007, 10.1584/jpestics.d18-007 PMID - 30479549 (2018). 10.1584/jpestics.D18-007 PMC6240775 30479549

[18] Gao YF (2021). Geographical and interspecific variation in susceptibility of three common thrips species to the insecticide, spinetoram. J. Pest Sci..

[19] Rotenberg D, Jacobson AL, Schneweis DJ, Whitfield AE (2015). Thrips transmission of tospoviruses. Curr. Opin. Insect Sci. Virol..

[20] Whitfield AE, Ullman DE, German TL (2005). Tospovirus-thrips interactions. Annu. Rev. Phytopathol..

[21] Ullman DE (2002). Thrips as vectors of tospoviruses. Adv. Bot. Res..

[22] Liu X (2023). Weak genetic structure of flower thrips *Frankliniella intonsa* in China revealed by mitochondrial genomes. Int. J. Biol. Macromol..

[23] Li H (2023). Chemosensory protein regulates the behavioural response of *Frankliniella intonsa* and *Frankliniella occidentalis* to tomato zonate spot virus-Infected pepper (*Capsicum annuum*). PLoS Pathog..

[24] Rotenberg D (2020). Genome-enabled insights into the biology of thrips as crop pests. BMC Biol..

[25] Catto MA (2023). Pest status, molecular evolution, and epigenetic factors derived from the genome assembly of *Frankliniella fusca*, a thysanopteran phytovirus vector. BMC Genomics.

[26] Guo SK (2020). Chromosome-level assembly of the melon thrips genome yields insights into evolution of a sap-sucking lifestyle and pesticide resistance. Mol. Ecol. Resour..

[27] Ma L (2023). Chromosome-level genome assembly of bean flower thrips *Megalurothrips usitatus* (Thysanoptera: Thripidae). Sci. Data.

[28] Zhang ZJ (2023). The chromosome-level genome assembly of bean blossom thrips (*Megalurothrips usitatus*) reveals an expansion of protein digestion-related genes in adaption to high-protein host plants. Int. J. Mol. Sci..

[29] Hu QL, Ye ZX, Zhuo JC, Li JM, Zhang CX (2023). A chromosome-level genome assembly of *Stenchaetothrips biformis* and comparative genomic analysis highlights distinct host adaptations among thrips. Commun. Biol..

[30] Zhang ZJ (2023). Chromosome-level genome assembly of the flower thrips *Frankliniella intonsa*. Sci. Data.

[31] Chen SF, Zhou YQ, Chen YR, Gu J (2018). fastp: an ultra-fast all-in-one FASTQ preprocessor. Bioinformatics.

[32] Kokot M, Dlugosz M, Deorowicz S (2017). KMC 3: counting and manipulating *k*-mer statistics. Bioinformatics.

[33] Ranallo-Benavidez TR, Jaron KS, Schatz MC (2020). GenomeScope 2.0 and Smudgeplot for reference-free profiling of polyploid genomes. Nat. Commun..

[34] Hu, J. *et al*. An efficient error correction and accurate assembly tool for noisy long reads. *bioRxiv*, 2023.2003. 2009.531669 (2023). 10.1186/s13059-024-03252-4 PMC11046930 38671502

[35] Walker BJ (2014). Pilon: an integrated tool for comprehensive microbial variant detection and genome assembly improvement. PloS ONE.

[36] Bolger AM, Lohse M, Usadel B (2014). Trimmomatic: a flexible trimmer for Illumina sequence data. Bioinformatics.

[37] Durand NC (2016). Juicer provides a one-click system for analyzing loop-resolution Hi-C experiments. Cell Syst..

[38] Dudchenko (2017). *De novo* assembly of the *Aedes aegypti* genome using Hi-C yields chromosome-length scaffolds. Science.

[39] Holst, F. *et al*. Helixer- *de novo* prediction of primary eukaryotic gene models combining deep learning and a hidden markov model. *bioRxiv*, 10.1101/2023.02.06.527280 (2023). 10.1038/s41592-025-02939-1 PMC13076211 41286201

[40] Mahram A, Herbordt MC (2015). NCBI BLASTP on high-performance reconfigurable computing systems. ACM Transactions on Reconfigurable Technology and Systems (TRETS).

[41] Song W, Wei SJ (2023). Figshare..

[42] Tarailo-Graovac, M. & Chen, N. S. Using RepeatMasker to identify repetitive elements in genomic sequences. *Curr. Protoc. Bioinformatics* Chapter 4, 1–14, 10.1002/0471250953.bi0410s25 (2009). 10.1002/0471250953.bi0410s25 19274634

[43] Flynn JM (2020). RepeatModeler2 for automated genomic discovery of transposable element families. Proc. Natl. Acad. Sci. USA.

[44] Gardner PP (2011). Rfam: Wikipedia, clans and the “decimal” release. Nucleic Acids Res..

[45] Burge SW (2013). Rfam 11.0: 10 years of RNA families. Nucleic Acids Res..

[46] Schattner P, Brooks AN, Lowe TM (2005). The tRNAscan-SE, snoscan and snoGPS web servers for the detection of tRNAs and snoRNAs. Nucleic Acids Res..

[47] Lagesen K (2007). RNAmmer: consistent and rapid annotation of ribosomal RNA genes. Nucleic Acids Res..

[48] (2023). NCBI Sequence Read Archive.

[49] (2023). NCBI Sequence Read Archive.

[50] (2023). NCBI Sequence Read Archive.

[51] (2024). NCBI GenBank.

[52] Simao FA, Waterhouse RM, Ioannidis P, Kriventseva EV, Zdobnov EM (2015). BUSCO: assessing genome assembly and annotation completeness with single-copy orthologs. Bioinformatics.

[53] Li H, Durbin R (2010). Fast and accurate long-read alignment with Burrows-Wheeler transform. Bioinformatics.

